# Data on initial leaf P concentrations and final dry matter yields of silage maize in response to row-injected cattle slurry

**DOI:** 10.1016/j.dib.2020.105570

**Published:** 2020-04-21

**Authors:** Ingeborg F. Pedersen, Gitte H. Rubæk, Tavs Nyord, Peter Sørensen

**Affiliations:** a Department of Agroecology, Aarhus University, Blichers Allé 20, PO box 50, 8830 Tjele, Denmark; b Department of Engineering, Aarhus University, Finlandsgade 12, 8200 Aarhus N, Denmark

**Keywords:** Cattle slurry, Inection Machinery, Maize (*Zea mays* L.), Nitrification inhibitor, Phosphorus balances, Placement, Row-injection, Slurry acidification

## Abstract

This article displays a dataset obtained in a field trial conducted in 2016 on a sandy loam and a coarse sandy soil, Denmark. Leaf phosphorus (P) and nitrogen (N) concentrations at the five-leaf stage (V5) and final dry matter (DM) yields of silage maize were determined in response to seven treatments with placed slurry below the maize row. Two row-injection methods combined with slurry acidification or addition of a nitrification inhibitor were tested. Furthermore final crop P uptake and P surplus at field level were determined.

This dataset can be used to assess the effect of placed slurry with or without slurry acidification and addition of a nitrification inhibitor on silage maize yields and to enhance our knowledge on maize P uptake and P surpluses at field level. In turn this can support the design of appropriate row-injection machinery of slurry.

The data supplied in this article is related to the research article entitled “Row-injected cattle slurry can replace mineral P starter fertiliser and reduce P surpluses without compromising final yields of silage maize” [1], where results from 2017 and 2018 are presented and discussed. The trials in 2016, 2017 and 2018 were conducted on the same study sites. The experimental design in 2017 and 2018 was a full-factorial design and did also include reference treatments with evenly injected slurry, whereas these reference treatments were not included in the present article.

Specifications table

**Table:** 

Subject	Agronomy and Crop Science
Specific subject area	Row-injection of cattle slurry in maize cropping
Type of data	Table Figure
How data were acquired	In-season sampling of youngest fully developed leaves and determination of final yields at harvest in a field experiment with maize
Data format	Raw Analyzed
Parameters for data collection	A field experiment was conducted in 2016 on two soil types; a coarse sandy soil and a sandy loam. Seven different treatments with placed slurry below the maize-row were tested, comprising combinations of the factors; placement method, slurry acidification and addition of a nitrification inhibitor to the slurry.
Description of data collection	The youngest fully developed leaves were sampled at the five-leaf stage. Maize plants were whole-crop harvested at silage maturity. hosphorus and nitrogen concentrations were determined in the leaves and in the biomass at the final harvest.
Data source location	Foulum and Havris, 8830-Tjele, Denmark Foulum: (56°49′ N, 9°56′ E) Havris: (56°53′ N, 9°41′ E)
Data accessibility	With the article
Related research article	Ingeborg F. Pedersen, Gitte H. Rubæk, Tavs Nyord and Peter Sørensen. Title: “Row-injected cattle slurry can replace mineral P starter fertiliser and reduce P surpluses without compromising final yields of silage maize” Journal: European Journal of Agronomy

## Value of the data

• The data is useful to assess the effect of different slurry row-injection techniques on silage maize yields grown in humid temperate regions.
• Other scientist studying slurry management and maize cropping can benefit from these data, when dealing with studies on crop growth in response to placed slurry. Furthermore, phosphorus crop uptakes and balances are provided, and these data can be used to assess crop phosphorus demands and phosphorus accumulation in soil.
• The data can be used to develop appropriate slurry-injection machinery in order to improve the utilization of slurry nutrients.
• The data offers information on maize yield in two additional trials conducted in 2016 related to [1], where data from 2017 and 2018 are presented.

## 1 Data description

### 1.1 Soil properties and weather data

Table 1 presents soil properties for each field at the two experimental sites in 2016. Monthly precipitation and temperature are provided for 2016 at the two study sites (Table 2).

**Table 1:** Soil properties at the two experimental sites.

Soil properties	Foulum	Havris
Soil texture	Sandy loam	Coarse sand
Clay (<2 µm), g 100 g^−1^ soil	8.3	4.2
Silt (2–20 µm), g 100 g^−1^ soil	7.8	2.7
Fine sand (20–200 µm), g 100 g^−1^ soil	49.7	32.4
Coarse sand (200–2000 µm), g 100 g^−1^ soil	34.1	60.8
pH (0.01M CaCl_2_)	5.9	5.8
Bicarbonate-extractable P, mg kg^−1^ soil^a^	49	34
Soil organic carbon, g 100 g^−1^ soil	1.6	1.5
NH_4_-N in 0-75 cm depth, kg ha^−1^	13.8	17.5
NO_3_-N in 0-75 cm depth, kg ha^−1^	32.1	35.0

a modified after Banderis, Barter [2].

**Table 2:** Cumulative monthly precipitation at 1.5 m height and mean monthly air temperature at 2 m height in the experimental period in 2016 including long-term mean (1961-1990) No data were available in March, April and September at Havris.

Month	Precipitation (mm)			Temperature (˚C)		
	Foulum	Havris	Long-term mean	Foulum	Havris	Long-term mean
March	24.8	n.a.	41	3.5	n.a.	1.8
April	101.4	n.a.	35	5.9	n.a.	5.5
May	41.7	29.1	45	12.8	13.0	10.5
June	109.4	70.2	52	15.6	15.8	14.2
July	97.9	86.6	67	15.9	16.3	15.4
August	65.6	72.7	66	15.5	15.9	15.1
September	16.7	n.a.	69	15.6	n.a.	12.1
October	78.6	32.3	68	8.5	8.1	8.5

### 1.2 Slurry properties, overview of treatments and main field operations

Overview of the treatments and their abbreviations are presented in Table 3. Slurry properties and slurry application rates are provided in Table 4, and dates for main field operations for 2016 are given in Table 5.

**Table 3:** Treatment overview showing experimental combinations of slurry application method, nitrification inhibitor (NI), slurry acidification (SA) and mineral starter N and P application. GF: Goosefoot tine with a 26-cm broad tine at 10 cm or 17 cm depth with a tine distance of 75 cm. S-spring tine: 6-cm wide S-spring tine at 10 cm depth with a tine distance of 37.5 cm.

Abbreviation	Slurry application method	Nitrification inhibitor (L ha^−1^)	Slurry acidification
NB untreated	Narrow band (NB) row-injection with S-spring tine, 10 cm depth	0	No
NB+SA	Narrow band (NB) row-injection with S-spring tine, 10 cm depth	0	Yes
NB+NI	Narrow band (NB) row-injection with S-spring tine, 10 cm depth	2	No
BB untreated 17 cm	Broad band (BB) row-injection with GF tine, 17 cm depth	0	No
BB untreated	Broad band (BB) row-injection with GF tine, 10 cm depth	0	No
BB+SA	Broad band (BB) row-injection with GF tine, 10 cm depth	0	Yes
BB+NI	Broad band (BB) row-injection with GF tine, 10 cm depth	2	No

**Table 4:** Cattle slurry properties and application rates in 2016.

Slurry properties and application rate	
DM content, %	7.9
Total N, kg Mg^−1^	3.8
NH_4_^+^-N, kg Mg^−1^	2.2
Total P, kg Mg^−1^	0.70
Total K, kg Mg^−1^	3.9
pH in untreated slurry	6.9
pH in acidified slurry	4.9
Slurry application rate, Mg ha^−1^	45
Slurry P application rate, kg ha^−1^	31.8
Slurry N application rate, kg ha^−1^	173
Total N application^a^, kg ha^−1^	263

a Total N application rate=slurry N+starter mineral N+later surface N application.

**Table 5:** Dates of main field operations in 2016.

Field operation	Havris/Foulum
Previous crop	Grass clover/ Spring barley
Ploughing	April
Row-injection of slurry	11.05
Sowing + Starter mineral N	17.05
Chemical weed control	06.06
	Callisto (0.5 L ha^−1^)
	MaisTer (50 g ha^−1^)
	MaisOil (0.67 L ha^−1^)
Leaf sampling at V5	13.06
N fertilization (70 kg N ha^−1^)	21.06/21.06
Chemical weed control	22.06
	Starship (0.5 L ha^−1^)
	MaisTer (50 g ha^−1^)
	MaisOil (0.67 L ha^−1^)
Whole-crop harvest	20.10/13.10

### 1.3 Initial leaf P concentrations and final dry matter yields of silage maize

Table 6 presents the P and N concentration at five-leaf stage (V5). At Foulum, the lowest leaf P concentration at V5 was observed when the broad-banded (BB) slurry was placed at 17 cm depth (BB untreated 17cm). The highest P concentrations were observed when a nitrification inhibitor was added to the slurry or when the slurry was acidified in combination with placement in narrow band (NB) or broad bands (NB+SA, NB+NI, BB+SA, BB+NI, Table 6). At Havris, the lowest leaf P concentrations at V5 were observed when the slurry was placed in broad bands at 17 cm depth (BB untreated 17cm). The highest leaf P concentration at V5 was observed when acidified slurry was placed in broad bands (BB+SA).

**Table 6:** Leaf phosphorus (P) and nitrogen (N) concentration at the five-leaf stage (V5). Different letters within columns denote statistically significant differences (Tukey, P < 0.05). NB: Narrow band injection, BB: Broad-band injection, SA: slurry acidification, NI: Nitrification inhibitor. The two highest P and N concentrations for each location are indicated by bold numbers.

Treatment	Leaf P concentration at V5, % of DM		Leaf N koncentration at V5, % of DM	
	Foulum	Havris	Foulum	Havris
NB untreated	0.30^b^	0.37^ab^	4.72^b^	4.72^a^
NB+SA	**0.37**^**a**^	0.37^ab^	**5.43**^**a**^	4.85^a^
NB+NI	**0.38**^**a**^	0.37^ab^	**5.26**^**ab**^	**4.97**^**a**^
BB untreated 17 cm	0.25^c^	0.33^b^	4.79^b^	4.80^a^
BB untreated	0.30^b^	0.35^ab^	4.97^ab^	4.86^a^
BB+SA	0.36^a^	**0.40**^**a**^	5.07^ab^	**5.02**^**a**^
BB+NI	0.36^a^	0.37^ab^	5.20^ab^	4.81^a^

Table 7 presents the DM yield and P uptake at harvest and the P surplus defined as P applied with cattle slurry minus P exported with the crop. At Foulum, treatments where slurry was placed in narrow or broad bands at 10 cm depth in combination with slurry acidification or a nitrification inhibitor (NB+SA, NB+NI, BB+SA and BB+NI) provided the highest DM yields. In these particular treatments, the DM yields were on average 1.5 Mg DM ha^−1^ higher than the DM yield observed in the treatment where slurry was placed in a broad band at 17 cm depth (BB untreated 17cm). At Havris, no significant treatment effect on DM yield and P uptake at harvest was observed.

**Table 7:** Maize dry matter (DM) yield, P uptake and P surplus at harvest for Foulum and Havris. Different letters within columns denote statistically significant differences (Tukey, *P* < 0.05). NB: Narrow band injection, BB: Broad-band injection, SA: slurry acidification, NI: Nitrification inhibitor. The two highest DM yields for each location are indicated by bold numbers.

Treatment	DM yield, Mg ha^−1^		P uptake, kg ha^−1^		P surplus, kg ha^−1^	
	Foulum	Havris	Foulum	Havris	Foulum	Havris
NB untreated	16.7^bc^	15.1^a^	35.8^a^	24.9^a^	-4.0^a^	6.9^a^
NB+SA	17.2^a^	15.2^a^	36.2^a^	28.3^a^	-4.4^a^	3.6^a^
NB+NI	17.2^ab^	15.2^a^	36.2^a^	28.3^a^	-4.3^a^	3.5^a^
BB untreated 17 cm	15.6^c^	15.1^a^	31.7^a^	28.9^a^	0.1^a^	2.9^a^
BB untreated	16.1^bc^	15.1^a^	33.4^a^	28.6^a^	-1.6^a^	3.2^a^
BB+SA	**17.7**^a^	**15.4**^a^	33.6^a^	28.0^a^	-1.8^a^	3.8^a^
BB+NI	**17.4**^a^	**16.4**^a^	36.1^a^	31.9^a^	-4.2^a^	-0.1^a^

**Fig. 1:**
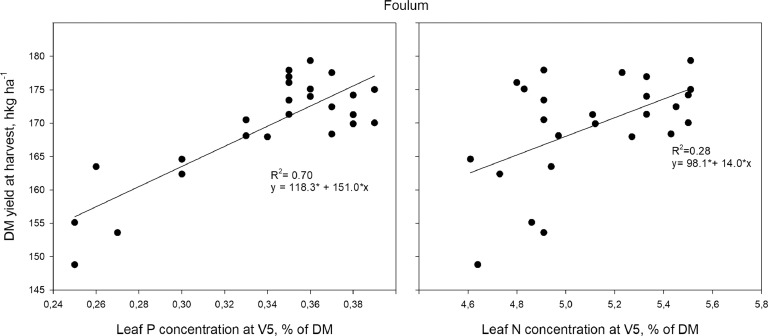
Leaf P and N concentrations at five-leaf stage (V5) plotted against dry matter (DM) yield at harvest at Foulum. The solid line represents the linear regression and asterisks (*) indicate significant slopes and intercepts (*P*<0.05).

**Fig. 2:**
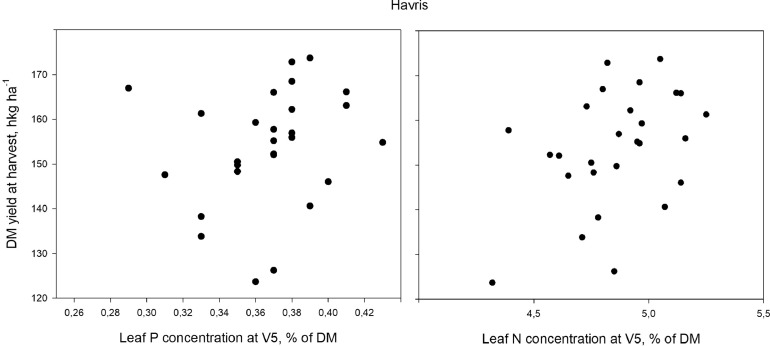
Leaf P and N concentrations at five-leaf stage (V5) plotted against dry matter (DM) yield at harvest at Havris.

Figs. 1 and 2 present the relation between leaf P and N concentrations and final DM yield at harvest for each of the experimental sites.

## 2 Experimental design, materials, and methods

### 2.1 Experimental design

A field experiment was established in 2016 on two soil types: a sandy loam at Foulum and a coarse sand at Havris in Central Jutland, Denmark (Table 1). The Foulum soil is classified as a Typic Hapludalf and the Havris soil as a Typic Haplorthod according to the USDA Soil Taxonomy System. The climate is temperate and humid (Table 2). The experiment was organized as a randomized block design with four replicates and seven treatments (Table 3). The plot size was 18 × 3 m (encompassing four rows), and the harvest plot size was 18 × 1.5 m (two middle rows).

### 2.2 Row-injection of slurry

Following ploughing, cattle slurry (Table 4) was row-injected at an application rate of 100 kg slurry-NH_4_^+^-N ha^−1^. Slurry was injected below the maize row with a 26-cm broad goosefoot tine with a tine distance of 75 cm (BB row-injection) at 10 or 17 cm depth from the soil surface to the bottom part of the slurry band, or with a 6-cm S-spring tine with a tine distance of 37.5 cm (NB row-injection). For treatments applied with acidified slurry, acidification was carried out in the slurry tanker by adding 13 L 7.08 *M* sulfuric acid (AcidLine^Ⓡ^, DanGødning, Fredericia, Denmark). For treatments receiving slurry with a nitrification inhibitor, 3.4-dimethylpyrazole phosphate (DMPP) was added in the slurry tanker as Vizura (BASF, Ludwigshafen, Germany) with an application rate of 2 L ha^−1^. Treatment overview is presented in Table 3.

20 kg mineral N ha^−1^ (as ammonium sulphate nitrate) was placed at the time of sowing in all treatments. In addition, a supplementary broadcast mineral N fertiliser dressing at a rate of 70 kg N ha^−1^ (as ammonium sulphate nitrate) was applied at the six-leaf stage in all treatments.

Maize (cv. Atrium FAO) was sown at 5 cm depth in early May with a 75-cm row spacing and 13.3 cm between plants within rows. Herbicides were applied on all plots. Field operations are listed in Table 5.

### 2.3 Sampling and analytical methods

At the five-leaf stage (V5), 40 of the youngest fully developed leaves were sampled manually in each harvest plot. The maize plants were whole-crop harvested at silage maturity leaving 15 cm stubble. The DM content was determined on a subsample of approximately 1 kg of the chopped fresh material. Plant material was oven-dried at 60 ˚C to constant weight (min 48 h).

Leaf P concentration was determined by digesting 1.5 g dried plant material in concentrated hydrochloric acid after ashing at 500 ˚C. The P concentration in the digest was determined by inductively coupled plasma-optical emission spectroscopy (ICP-OES, Yara, Analytical Services, Pocklington, UK). Leaf N concentration was determined by Kjeldahl digestion.

Phosphorus concentration of the whole crop was determined by digestion under pressure in a microwave oven following measurement by ICP-OES (EurofinsAgroTesting, Denmark).

### 2.4 Statistical analyses

Data from the two sites was analyzed in the R-Project software package version 3.4.1 using linear mixed-effects models from the R-package *lme4* with treatments as a fixed effect and replicate as a random effect. The assumption of homogeneity of variance and normality of residuals was visually verified by plot of residuals against fitted values and histogram of the residuals. In cases where the treatment effect was found to be significant in a one-way analysis of variance, the differences between treatments for each location were analyzed by the Tukey´s honestly significant difference (HSD) using estimated marginal means from the R-package *emmeans*.

Significance was declared at the P ≤ 0.05 level of probability.

## Appendix Supplementary material

Image, application 1

## References

[1] Pedersen I.F., Rubæk G.H., Nyord T., Sørensen P. (2020). Row-injected cattle slurry can replace mineral P starter fertiliser and reduce P surpluses without compromising final yields of silage maize. Eur. J. Agr..

[2] Banderis A., Barter D.H., Henderson K. (1976). The use of polyacrylamide to replace carbon in the determination of “olsen's” extractable phosphate in soil. J. Soil Sci..

